# P-936. Impact of an Antimicrobial Susceptibility Reporting Change for Serratia marcescens

**DOI:** 10.1093/ofid/ofaf695.1139

**Published:** 2026-01-11

**Authors:** Kaytlin A Bishop, Elizabeth Neuner, Christine R Lockowitz, Tamara Krekel, David J Ritchie, Emily C Welch, Taryn B Mondiello, David A Rosen, Rebekah Dumm, Tristan T Timbrook

**Affiliations:** Barnes-Jewish Hospital, Saint Louis, MO; Barnes-Jewish Hospital, Saint Louis, MO; St. Louis Children’s Hospital, Trenton, Illinois; Barnes-Jewish Hospital, Saint Louis, MO; Barnes-Jewish Hospital, Saint Louis, MO; Barnes Jewish Hospital, Saint Louis, Missouri; Barnes Jewish Hospital, Saint Louis, Missouri; Washington University School of Medicine, Saint Louis, Missouri; Washington University School of Medicine, Saint Louis, Missouri; Barnes-Jewish Hospital, Saint Louis, MO

## Abstract

**Background:**

­The use of labile, weak AmpC-inducing β-lactams (e.g., ceftriaxone (CRO), ceftazidime (CAZ), piperacillin-tazobactam (TZP)) for organisms with moderate risk of inducible AmpC production may lead to treatment failure due to AmpC overproduction. Recent guidance from the Infectious Disease Society of America and Clinical Laboratory Standards Institute state *Serratia marcescens* is at low risk of significant AmpC production and suggests it is reasonable to treat low-risk organisms with a labile, weak AmpC-inducing β-lactam. Prior to October 2023, *S. marcescens* isolates were reported as resistant to CRO, CAZ and TZP at our institution; these susceptibilities are now reported as tested.

**Figure 1.**
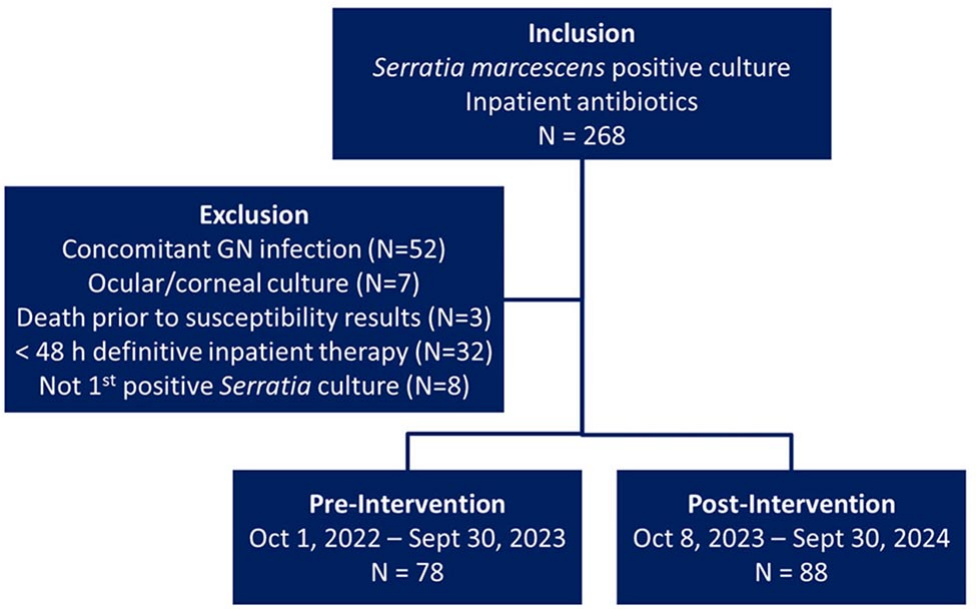
Inclusion-Exclusion Flowchart

**Table 1.**
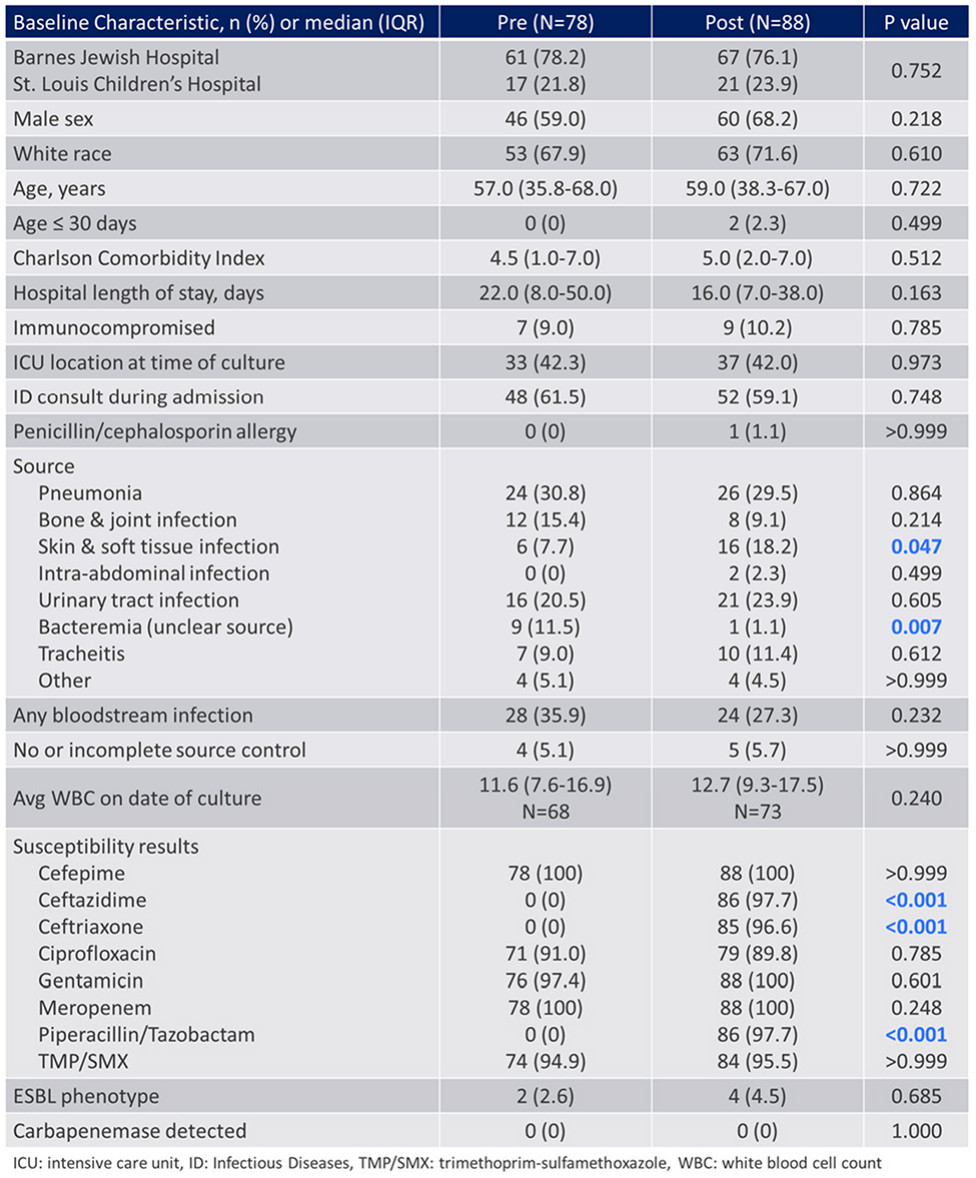
Baseline Characteristics

**Methods:**

This study evaluated safety, effectiveness, and antibiotic utilization before and after this change in susceptibility reporting. The primary outcome was AmpC stable β-lactam (cefepime or carbapenem) inpatient days of therapy (DOT). This pre-test (October 2022–September 2023), post-test (October 2023–September 2024) quasi-experiment included inpatients at Barnes-Jewish and St. Louis Children’s Hospitals with first-positive *S. marcescens* culture and at least 48 hours of antibiotic therapy. Exclusions included concomitant Gram-negative infections resistant to CRO, CAZ, or TZP, ocular cultures, and death prior to susceptibility results.

**Table 2.**
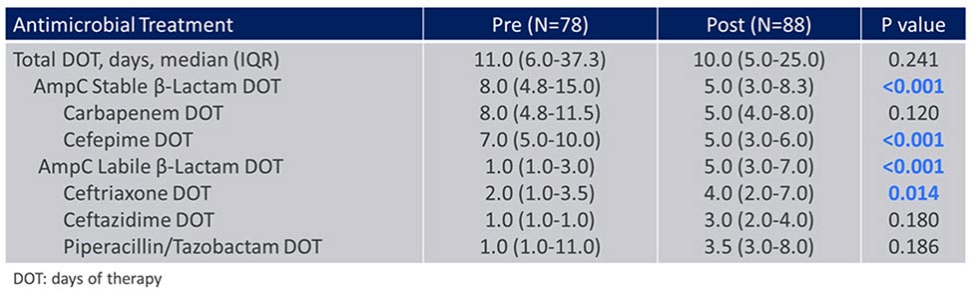
Days of Gram-Negative Antimicrobial Therapy

**Table 3.**
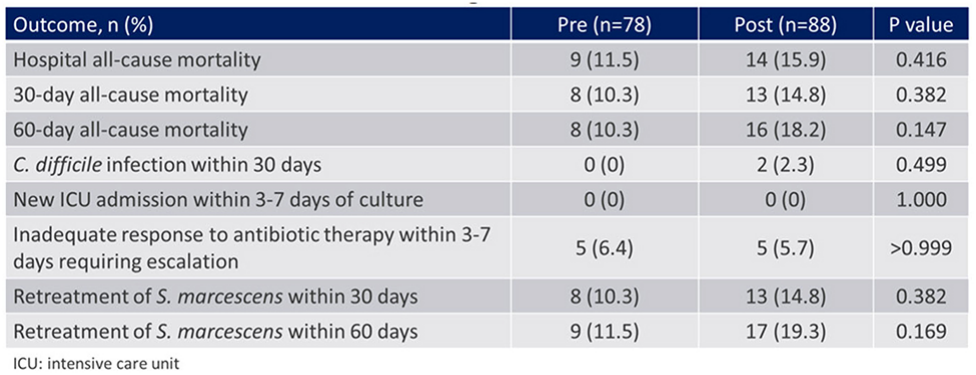
Clinical and Microbiological Outcomes

**Results:**

A total of 268 patients were screened and 166 patients were included (Figure 1). Baseline characteristics including age, ICU status, immunocompromised status, and no or incomplete source control were well balanced between groups (Table 1). The most common infection source was pneumonia (30%). The median total DOT for AmpC stable β-lactams was 8 in the pre-group vs 5 in the post-group (P < 0.001) (Table 2). In patients treated with ≥ 3 days of AmpC labile β-lactams who had repeat cultures, there was no emergence of resistance (0/26). No statistically significant differences in clinical outcomes were observed (Table 3).

**Conclusion:**

In conclusion, a change in susceptibility reporting for *S. marcescens* was associated with a decrease in AmpC stable β-lactam use, with no significant changes in microbiological or clinical outcomes.

**Disclosures:**

Christine R. Lockowitz, PharmD, BCIDP, AbbVie: Grant/Research Support|Premier Inc.: Honoraria Tamara Krekel, PharmD, BCPS, BCIDP, AbbVie: Advisor/Consultant|AbbVie: Honoraria|Shionogi: Advisor/Consultant|Shionogi: Honoraria David J. Ritchie, PharmD, BCPS (AQ-ID), Shionogi: Honoraria Rebekah Dumm, PhD D(ABMM), BD: Advisor/Consultant|BD: Grant/Research Support|Biomerieux: Advisor/Consultant|Biomerieux: Grant/Research Support|Diasorin: Grant/Research Support|Pattern Biosciences: Grant/Research Support|Qiagen: Grant/Research Support|Roche Diagnostics: Advisor/Consultant|Shionogi: Advisor/Consultant

